# Beyond health economics: economic growth, artificial intelligence, and environmental externalities in shaping life expectancy

**DOI:** 10.3389/fpubh.2026.1785053

**Published:** 2026-03-31

**Authors:** Mohammed Moosa Ageli

**Affiliations:** Department of Economics, College of Business Administration, King Saud University, Riyadh, Saudi Arabia

**Keywords:** AI, environmental sustainability, GCC countries, health economics, life expectancy, system GMM approach

## Abstract

**Introduction:**

Life expectancy is determined by several structural interplays. This study examines the impact of economic growth, AI (as measured by digitalization), and carbon emissions on life expectancy in GCC countries from 2000 to 2024.

**Methods:**

This research relies on a standard model derived from the health economics function, which combines panel fixed effects and dynamic models (System GMM) to measure the impact of economic growth, digital transformation, and environmental sustainability on public health. Estimates were obtained using static fixed effects and a dynamic specification corresponding to the generalized multiple methods approach. These estimates are useful for controlling for unobserved heterogeneity, health persistence, and possible internal relationships among income, technology, and health. Life expectancy indicates a high level of persistence.

**Results:**

The dynamic estimates indicate that the lag values of the current health outcome can explain the outcome over 70 years. After controlling for static and dynamic specifications and internalities, the economic growth role is positive but reduced. All specifications indicate that digital transformation has a positive and robust effect on life expectancy, with an economically significant magnitude. In contrast, a strong correlation exists between per capita carbon dioxide emissions and low life expectancy. The proportion of health expenditure to GDP is not significantly associated with life expectancy in the GCC countries.

**Discussion:**

Overall, these findings imply that population health improvements in GCC countries are probably driven less by health expenditure volumes and more by structural changes in the digital and environmental efficiency of population health production. Economic development will expand fiscal space for health-sector expenditures. The development of the digital economy clearly increases the productivity of health sector expenditures. Conversely, carbon-intensive growth imposes significant health costs. The findings elucidate that digital and environmental health organizations within the public health sector are essential for achieving health gains and long-term sustainability in GCC countries.

## 1 Introduction

Sustainable development in high-income, resource-dependent economies poses a structural paradox: historically, economic expansion relies on energy-intensive growth models that generate environmental externalities that can undermine long-term social welfare.

The link between AI (in terms of digital transformation) and sustainable development, economic growth, and public health in the GCC is an integrated economic framework that can be read as a self-feeding productive, health, and technical cycle: growth expands the fiscal and investment space of the state and the private sector, sustainability reduces resource efficiency and reduces environmental and community risks (1).

AI raises the marginal productivity of capital and labor, reduces transaction costs, and increases the accuracy of resource allocation, whereas improvements in public health are due to improvements in human capital quality, productivity, and the reduction of workday loss, thereby raising the overall productivity of the factors of production (2).

This cumulative cycle is clearly shown in GCC countries because they simultaneously combine the characteristics of high-income economies, rapidly transformed urban communities, expandable public/mixed health systems, advanced digital structures, and environmental and climate risks with direct economic weight, making the region’s health economies more sensitive to the interaction between technology and sustainability than many other regions (3).

Data on life expectancy at birth from 2000 to 2023 show that GCC countries have undergone a gradual health transition, with inequalities between countries, and that the COVID-19 trauma is reflected in short-term fluctuations followed by a strong rebound later (4).

In Saudi Arabia, life expectancy increased from 72,480 years in 2000 to 77,971 years in 2023 and then declined to 74,217 years in 2021. In the UAE, it increased from 76,347 years in 2000 to 82,909 years in 2023, with a marked decline in 2021 to 79,083, followed by a recovery (5).

In Bahrain, it rose from 74,673 years in 2000 to 81,284 years in 2023, then declined to 78,683 in 2020 and recovered. This pattern (long-term improvement with short-term disorder in 2020–2021) reflects three elements from the perspective of health economics: (1) progress in prevention, coverage, control of chronic diseases, traffic safety improvements, and primary care over two decades; (2) short-term vulnerability to epidemiological shocks; and (3) the ability of health supply and governance systems to recover when resources, data, and operational flexibility are available (6).

Because AI, in particular, enhances the capacity for rapid, data-driven responses, the economic value of AI in health extends beyond increasing hospital efficiency to reducing losses from trauma-related health system disorders (7, 8). As AI is a general-purpose technology that requires digital infrastructure and high communication to be translated into cost savings or quality gains at a population scale, digital use indicators serve as a mediating variable between economic growth and its health impact.

In Saudi Arabia, the Internet user index (as a percentage of the population) increased from 21.07% in 2000 to 100% in 2021, a structural transformation over two decades from an information economics perspective. According to the same series, it increased from 4.8637% in 2000 to 99.6528% in 2021 (9), indicating a rapid transition to semi-digital use.

At the latest level published in other series of the same indicator (such as 2023 summaries), Saudi Arabia appears at 100% in 2023, indicating continued digital (10, 11).

These numbers not only provide my communication but also economically indicate a significant reduction in the cost of access to health information, an expansion in the digital health services market, higher possibilities for communication medicine, telemedicine management, increased pharmaceutical obligations, improved health education, and reduced information inequality between the service provider and the patient, all known channels in health economics to improve health outcomes at a lower marginal cost (12, 13).

However, GCC states face a significant health economic paradox: high incomes in rent/semi-retail economies may be associated with high risk factors for non-communicable diseases, such as obesity, diabetes, and hypertension, due to rapid urbanization and consumption patterns, which means that growth at the same time may raise the demand for expensive treatment services if prevention and behavioral governance tools are not activated (14, 15).

Sustainability and AI work together: sustainability through health city policies, active transport, air quality, food security, and water management; AI by predicting individual and population risks, allocating prevention interventions, and improving evidence-based health program design (16–18).

**Figure 1:**
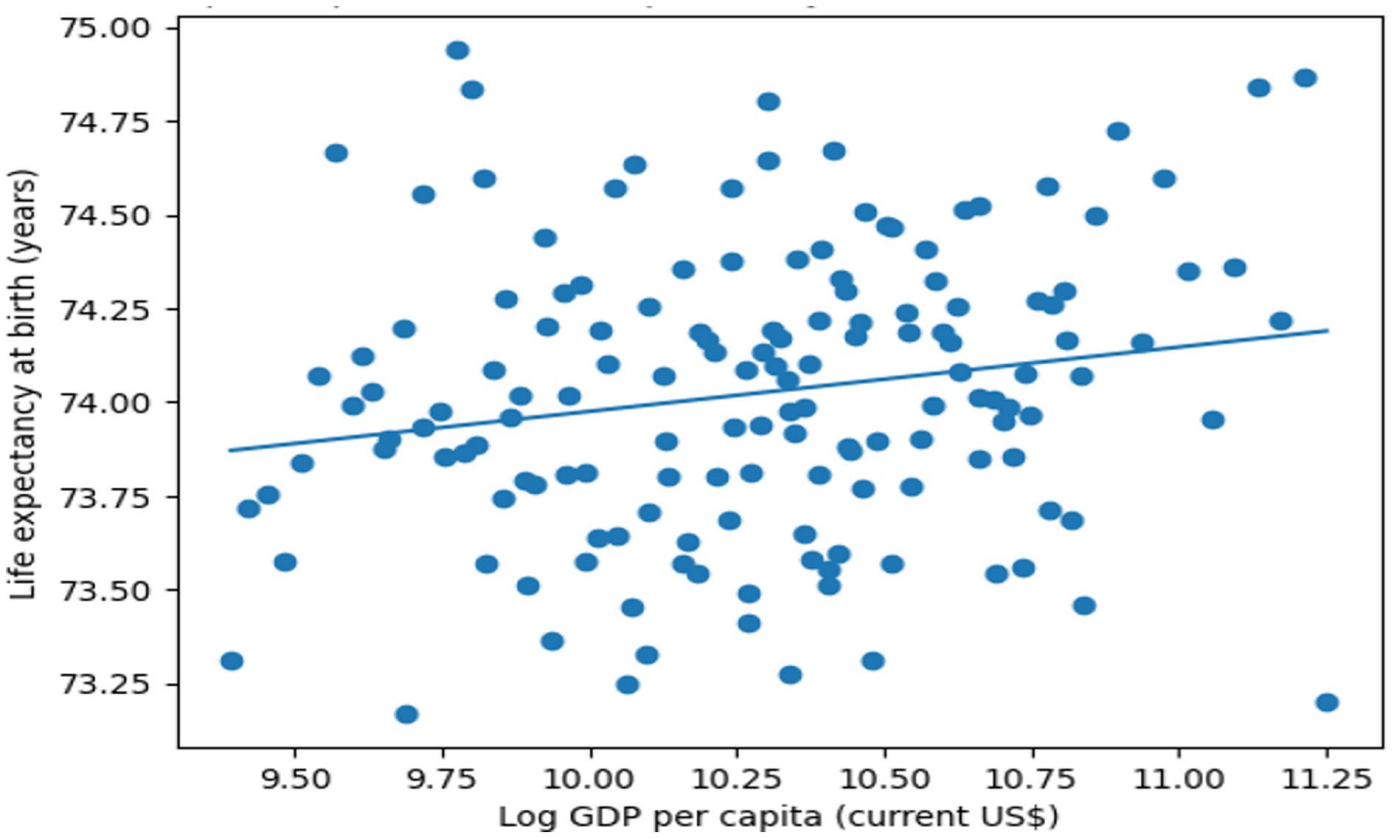
GDP per capita and life expectancy in developing countries.

Figure 1 shows that each point represents a country–year observation from the World Bank’s Index of Development Indicators. The solid line shows the fitted linear regression of life expectancy at birth on log GDP per capita. The figure illustrates the positive income–health gradient observed in GCC countries during the study period.

While higher income levels are associated with greater life expectancy, the relatively moderate slope suggests diminishing marginal health gains at higher income levels. The scattered points around the fit curve indicate heterogeneity in the relationship, which may be due to factors other than income. The use of carbon emissions and digitalization as structural drivers of more adequate variations motivates the subsequent econometric analysis that accounts for environmental externalities.

From a macroeconomic perspective of public health, the relationship among the three variables (AI, sustainability, and growth) can be summarized by the following simplified health production equation: population health is a function of real income per capita, environmental quality, health system efficiency, human capital, governance, and the spread of technology (19, 20).

During 2000–2023, the increases in life expectancy in Saudi Arabia, the Emirates, and Bahrain can be attributed to a cumulative product of these factors, with relative weights varying across countries (5, 21). In 2023, the Emirates recorded a projected age of 82,909 years, up from 76,347 in 2000, representing a gain of approximately 6,562 years. Saudi Arabia recorded an increase of approximately 5.491 years, from 72.480 in 2000 to 77.971 in 2023 (22). Bahrain recorded an increase of approximately 6.611 years, from 74.673 in 2000 to 81.284 in 2023 (5, 21).

Sustainability intersects with public health in the GCC states through the lens of environmental risks, which are increasing economic burdens as climate change intensifies. High temperatures, humidity, dust, and repeated heat waves increase the burden of respiratory and heart diseases, increase occupational exposure risk, and reduce productivity in exposed sectors, creating indirect economic costs to output and health expenditure.

At this point, investments in sustainability, such as energy efficiency, urban greening, air quality, waste management, and public transport, are as financially sound as they are environmentally beneficial (23–25).

Here, AI works as an enabling layer of the sustainability economy by improving local weather forecasting and air quality, efficiently managing electricity and water networks, strengthening pharmaceutical supply chains under climate risk, and identifying healthier areas and groups to guide interventions. Economically, this means that part of sustainability’s “health return” is reflected not only in disease and death indicators but also in reduced production fluctuations, lower disruption costs, and reduced pressure on emergency facilities during dust and extreme heat seasons. In 2023, the UAE reached an expected life expectancy of 82 years (26).

The period from 2000 to 2024 in the GCC states can be described as a transition from an expansionary care economy to a data-based care economy, with varying speeds of transition. Indicators of life expectancy show that progress has already been achieved in public health outcomes between 2000 and 2023, suggesting that health production has improved despite high levels of chronic risk factors (27).

In Saudi Arabia, internet penetration, which rose from 2.2107% in 2000 to higher levels, has changed the economics of caregiving by expanding the market for communication, digital follow-up services, and behavioral health programs on a large scale, which can have a critical impact on chronic diseases that require long-term commitment (28).

In Qatar, a relatively higher start in 2000 (4.8637%) and acceleration to 99.6528% in 2021 reflect the high adoption of advanced digital care models in a relatively minor health market, which facilitates experimentation with system-wide AI solutions and their rapid dissemination (29, 30).

In the UAE, the life expectancy reached 82,909 years in 2023. This indicates that the health system has achieved outputs close to those of advanced economies, which makes it possible to maximize the health value of every dirham (AED) through techniques that reduce marginal costs, increase prevention, and lower the price of avoidable hospitalizations (31, 32).

In 2023, Bahrain ‘s total revenue was BHD 81,284, demonstrating that a mix of health policies, urban structure, and service effectiveness has achieved high revenue, and that AI can support financial sustainability by managing demand and improving population health planning.

Sustainability is a structural driver of health demand: better air quality, lower pollutant emissions, and advances in food safety and city planning policies lead to a decline in demand for some expensive treatment services and higher health expenditure efficiency through lower preventable cases (33, 34).

Economically, investment in sustainability can be justified as a healthy investment with an indirect internal return: reducing the costs of disease and productivity loss, as well as environmental returns. Artificial intelligence maximizes this return by improving the targeting of investments and policies: instead of costly public solutions, accurate risk maps can be built and interventions directed to neighborhoods and the most vulnerable groups, thereby raising the marginal return of each unit of public expenditure (35).

Hence, the adoption of AI in public health becomes economically convincing when (1) it reduces the condition or therapeutic package’s expected cost, (2) it improves health outcomes to justify the additional cost according to the approved willingness to pay, or (3) it achieves both (36). In the GCC states, the environment is highly saturated, and life expectancy continues to improve.

The largest gains will often be in chronic disease management and prevention, increasing the health workforce’s productivity, adjusting supply chains, and improving the spatial allocation of services, because these areas carry the most significant long-term cost burden (37).

Moving to 2025 as the time horizon in the study’s title, recent economic readings distinguish between digitization and AI: digitization is about service delivery and data recording, while AI is about transforming data into decisions, customization, forecasting, and automation (38, 39). The challenge until 2025 and beyond is not to expand communication as much as to raise data quality, standardize standards, establish governance rules for the use of health data, link financing to measurable results, and manage the risk of bias and privacy (40, 41).

From an economic perspective, the research contribution of this study is that it transmits the analysis of public health in GCC countries from their traditional framework as an expenditure burden to their modern framework as a productive origin and human capital, and redefines AI and sustainable development as tools for raising economic efficiency rather than technical or environmental orientations. Instead of considering each axis in isolation, this study offers an integrated perspective that explains how economic growth affects health demand and how sustainability and artificial intelligence (AI) shift this demand from escalating therapeutic costs to low-cost, high-yield preventive investments.

The research also focuses on the economic value of public health, not just the size of expenditure; it shows that the real return in the GCC countries is achieved by improving their allocation through AI and linking financing to health outcomes, and by integrating sustainability policies as indirect public health policies that reduce the external costs of pollution and heat and support long-term productivity.

It has high applied value because it provides a framework for decision-makers to assess technical and sustainability policies together based on their health and economic impacts. It places public health at the heart of growth and economic diversification strategies rather than on the margins of fiscal policy.

This study addresses digitalization as a structural economic transformation (not directly through AI). In high-income economies, digital infrastructure enhances resource allocation efficiency, reduces transaction costs, and increases service productivity. Therefore, digital connectivity is an enabler of broader technological and institutional modernization on the sustainable development pathway.

In this context, population health is a crucial entry point for achieving sustainable development pathways. This study examines the impact of economic growth, digitalization, and carbon emissions on life expectancy in GCC countries from 2000 to 2024. This research relies on a standard model derived from the health economics function, which combines panel fixed effects and dynamic models (System GMM) to measure the impact of economic growth, digital transformation, and environmental sustainability on public health.

This study contributes to the literature on sustainable development in three ways by linking environmental externalities and digital capabilities as predictors of structural health in the production function. The study: (i) presents region-specific evidence from high-income carbon-intensive economies, measures the relative importance of structural versus fiscal health drivers, and unfolds the welfare implications of decarbonization and digital transformation for long-run development pathways.

Section 2 presents a comprehensive literature review, establishes a theoretical framework, and outlines a research gap. The methodology and data used in this study are described in Section 3. Section 4 presents the results of the GMM test for the model and system. Section 5 presents the discussion. Section 6 presents the recommendations, limitations, and policies.

## 2 Literature review

The study of economic, technological, and environmental determinants of public health has seen a marked expansion in the literature on health economics, with a clear transition from static analysis to dynamic models that address the continuity of health and the use of reverse causal indicators. This shift reflects an increasing awareness of public health as a cumulative asset shaped by long-term economic and technological trajectories rather than short-term shocks (42).

Recent studies indicate that the positive relationship between GDP per capita and life expectancy continues, especially in middle-income countries, but with a clear emergence of declining marginal returns.

Chen et al. (43) evaluated the key drivers of spatial access to health services, highlighting the important roles of the spatial distribution of the population, health resources, and transport infrastructure in enhancing it. Chishui County, a national-level poverty-stricken county from Guizhou province, China, was chosen as our study area. The study, conducted from 2014 to 2017, used an enhanced two-step floating catchment area (E2SFCA) to examine spatial access to health services using geographic information system (GIS) data and the time–distance between the population and sanitation.

Chain substitution was also used to determine each factor’s contribution to changes in access levels. Increasing health resources, such as the number of medical facilities and health staff, was the most influential factor affecting access to health care. Variations in characteristics can result in different outcomes. Therefore, better health care access requires increasing health resources, planning transport, and distributing services according to population distribution.

Studies based on OECD countries from 1996 to 2020 showed that economic growth improves public health through indirect channels, such as higher living standards and improved health infrastructure. However, this impact weakens when institutional and technological factors are controlled (44, 45).

Studies have also indicated that if health continuity is not considered, static estimates tend to overstate the impact of income (46–49).

Regarding healthcare quality and medical expenses, Xue et al. (50) analyzed the performance of public, private, and nonprofit hospitals in Sichuan County, China. They utilized patient data on 64,171 admitted patients with pneumonia, heart failure, and AMI from late 2016 to 2018. Ownership-multilevel regression was conducted on mortality and medical expenses. According to the study, the mortality rate from pneumonia was higher in private and nonprofit hospitals than in public hospitals.

Similarly, no difference was observed in the rates of heart failure and infarction. The infarction costs did not vary by hospital ownership; however, the costs of pneumonia and heart failure were higher in private hospitals. Overall, public hospitals deliver care that is comparably effective or superior at a lower cost than private hospitals. Little difference was found between private and nonprofit hospitals.

Given these problems, dynamic models, especially system-generalized multiple models (GMMs), have been increasingly used to analyze the relationship between economic growth and health. Studies from 1990 to 2022 that included samples from OECD countries, China, and Africa from 1990 to 2022 showed that the time-lagged variable (life expectancy or infant mortality) was high and statistically significant, confirming the cumulative nature of public health and limiting the immediate impact of economic growth. These results are systematically consistent with the approach adopted in this study (51–53).

Since 2020, digital transformation has become a central focus of public health research. Some studies indicate a positive relationship between the spread of Internet use and life expectancy in large international samples during 1997–2020, in which digitization operates through improved access to health information, expanded telemedicine services, and more efficient health system management (54, 55). However, most of these studies adopted static or partial causal models.

In return, more recent studies that use fixed effects and the system GMM approach have provided more stringent evidence that digitization continues to have a positive effect on public health, even after controlling for technology and health (56, 57). Studies from 2002 to 2019 on developing and emerging countries have shown that digital transformation not only improves life expectancy but also reduces health inequality, thereby enhancing the overall impact of technology on public health (58). These results are particularly important because they adopt the same standard dynamic strategy.

Regarding environmental sustainability, there is a broad consensus on the negative impact of CO2 emissions on public health. According to Hu et al. (59), green finance helps construct urban green technology transfer networks, which expedite the low-carbon industrial transformation of cities in the Yangtze River Delta, China.

Studies that have adopted a two-step System GMM Approach on international or regional samples during 2000–2020 have confirmed that even after controlling for income and health expenditure, high emissions are associated with lower life expectancy (60, 61). Studies focused on GCC countries during 1990–2020 also showed that their energy-intensive growth pattern generates tangible external health costs, making the relationship between growth and health more complex than in traditional industrial economies (24, 62, 63).

According to Hu et al. (64), pollution in China’s Yangtze River Delta harms public health and intensifies spatial health inequalities. Drawing studies have drawn from environmental administrative penalty data for the period 2012–2023. This study explores the structural features and factors influencing pollution transfer. The findings demonstrate that network structures have evolved into two bound states or more diverse states. In contrast, it strengthens the pollution transfer across other proximity. It also has a spatial health inequality pattern.

In contrast, some studies, especially those that use homogeneous samples of low-income states, suggest a positive link between emissions and life expectancy, as emissions reflect an early stage of manufacturing and improved basic services (65). These results are often explained in terms of different stages of development and do not necessarily conflict with negative results in high-income, emissions-intensive economies.

Health expenditures show different results. Some studies that have used the system GMM approach on OECD data have found that government health expenditures raise life expectancy and reduce infant mortality, whereas other studies have shown that when controlling for environmental and technological factors, this impact becomes statistically weaker or insignificant in high-income countries (66–68). This variation suggests that the effectiveness of health expenditures depends more on their efficiency and composition than on their size.

In general, the literature since 2020 reveals a systematic and substantive direction that aligns with the design of this study. Most studies have adopted panel fixed effects and the system GMM approach models to address and maintain endogeneity and persistence in health indicators and have concluded three central results: public health is a cumulative dynamic variable, digital transformation has become a key determinant of health in modern economies, and environmental degradation imposes a large external health cost that cannot be compensated for by growth or expenditure alone.

This study adds to the literature by providing a modern, dynamic guide that focuses on GCC countries through 2024, a region with limited research on global health economics.

### 2.1 Research gap

Despite the significant expansion of the recent literature on the determinants of public health, a systematic and applied gap persists, especially in high-income, energy-intensive economies, such as those in the GCC. Most studies have focused on the impact of economic growth or health expenditures on public health, independent of digital transformation and environmental sustainability, or have partially addressed these factors without an integrative framework that reflects their common interaction within the health-production function.

In addition, many studies rely on static models that do not account for the cumulative nature of public health, endogeneity, and adverse causality between health, growth, and technology, leading to biased and overstated estimates of the real-time impact of economic variables. Studies that use modern dynamic panel methods are limited in their application to the Gulf countries and often end at shorter time horizons that do not capture the accelerated digital and sustainable transformations since 2015.

Thus, an integrated, modern, and dynamic analysis that jointly assesses the impact of economic growth, digital transformation as an integrator of AI, and environmental sustainability on public health in GCC countries over an extended time frame is lacking.

This study closes this gap by employing panel fixed effects and system GMM approach models on recent data through 2024, thereby providing a more accurate understanding of the structural determinants of public health in a special economic and developmental context that has not been adequately considered in the existing literature.

Moreover, this study provides timely and policy-relevant answers to questions at the intersection of growth, digitalization, sustainability, and health. The proposed framework is conceptually quite appealing. The idea of an integrated theoretical framework that comprises these dimensions is quite appealing.

The use of both static and dynamic panel models is methodologically ambitious. Openly reported diagnostic tests for GMM. The focus on the region highlights the gap in the existing literature and its appeal, given the SDG and Vision 2030 policy narratives. Digital transformation and environmental externalities affect the health of the GCC population.

This study examines the impact of economic growth, digitalization, and carbon emissions on life expectancy in GCC countries from 2000 to 2024. This research relies on a standard model derived from the health economics function, which combines panel fixed effects and dynamic models (System GMM) to measure the impact of economic growth, digital transformation, and environmental sustainability on public health. In this study, Internet penetration is used as a proxy for digital transformation and technological readiness rather than as a direct measure of AI.

Although AI represents an advanced stage of digital capability, its diffusion requires widespread digital infrastructure and connectivity. Accordingly, the analysis conceptualizes digital transformation as a foundational structural enabler of productivity and health system efficiency, within which AI adoption may subsequently occur.

Moreover, this study situates health functions within a broader sustainable development paradigm, recognizing that health outcomes reflect the structural configuration of economic growth, technological capability, and environmental quality. In high-income, resource-dependent economies, sustainable development requires aligning growth regimes with environmental decarbonization and innovation capacity to internalize environmental externalities and enhance social welfare outcomes.

Public health is a form of cumulative human capital formed through the interaction of a set of economic, technological, and environmental variables that function over time. Health is instead a cumulative, long-run effect of both direct and indirect investments by individuals and society at large. Investments comprise income, technological advances, environmental quality, institutional, and demographic characteristics. Under this framework, economic growth can affect public health through various channels.

Economic growth improves living standards, increases the ability to pay for health services, and increases access to education, nutrition, and adequate housing. Health-producing function: Increased access to health-enhancing goods, services, and information. With technological advancements, digital transformation, and artificial intelligence (AI), the health-producing function has been incorporated. Furthermore, according to some economic theory, the marginal income effect may be declining in high-income economies.

Consequently, health gains from any additional dollar of income are probably limited unless efficiency and service quality improve. In exchange, digital transformation and artificial intelligence (AI) enhance the effectiveness of healthcare resources, reducing access costs and improving disease diagnosis, prevention, and management.

The theoretical framework holds that digitization is not only a growth complement but also an independent productive input that may engender health gains and stable incomes and expenditures. The framework includes environmental sustainability as a key dimension, connecting growth to health through the logic of external costs. Emissions from waste generated by growth are energy-intensive; hence, the net health impact of growth depends on the extent to which it reduces its negative environmental impact. In contemporary economies, environmental policies have become an important part of health policies.

The final assumption is that public health is continuous over time, implying that present behavior is largely determined by historical paths. Dynamical models should be used as a standard for analysis. This demonstrates the need for long-term policies that balance economic growth, digital transformation, and environmental sustainability.

## 3 Data and methodology

### 3.1 Data structure

This study adopts an econometric approach grounded in health economics to examine the impact of economic growth, digital transformation (as a proxy for AI readiness), and Internet usage (% of population), which captures digital access and infrastructure penetration. It does not directly measure AI implementation but serves as an indicator of technological readiness and system-level digital capacity within the broader digital transformation process, environmental sustainability, and its impact on public health outcomes in GCC countries from 2000 to 2024 (Table 1).

**Table 1:** Definitions, measurements, and data sources of the variables.

Variable name	Symbol	Measurement	Data source
Life Expectancy at Birth	LE	The average number of years a newborn is expected to live, assuming that current age-specific mortality rates remain constant.	World Bank–World Development Indicators
GDP per capita	GDPpc	Gross Domestic Product (GDP) in constant USD	World Bank–WDI
GDP growth rate	GDPg	Annual percentage change in real GDP	World Bank–WDI
AI (in terms of digital transformation)	DIG	% of individuals using the Internet.	World Bank–WDI
CO₂ Emissions per Capita	CO2	Metric tons of carbon dioxide emissions per capita per year.	World Bank: WDI and IEA
Share of renewable energy	REN	The share of renewable energy in total final energy consumption.	World Bank: WDI and IRENA
Health expenditure (percentage of GDP)	HEC	Current health expenditure as a percentage of gross domestic product (GDP)	World Bank – WDI; WHO
The population growth rate	POPG	Annual population growth rate.	World Bank–WDI
Urban population (percentage of the total population)	URB	Population percentage in urban areas	World Bank–WDI

The methodological framework is based on the concept of a health production function, in which population health is treated as an outcome of economic, technological, environmental, and institutional inputs.

Internet penetration is interpreted as a proxy for AI capability level, but rather as a proxy for digital foundational capacity, which denotes the presence of enabling factors that contribute to the proliferation of more advanced digital technologies, including AI applications. However, statistics alone do not proxy for AI penetration, usage levels, or the adoption of AI-capable technologies. The literature has recently begun to focus on measuring AI capabilities (69). To explore AI capabilities, multilayer measures (computer capacity, data governance, and AI in different applications).

Given the cumulative nature of health outcomes and the potential for endogeneity among key regressors, static panel estimators (fixed effects) are combined with dynamic panel techniques.

### 3.2 Methodology

The empirical analysis focuses on six GCC countries, reflecting the regional bloc’s institutional and structural coherence. Although the GCC has a relatively small cross-sectional dimension, it provides a policy-relevant and economically comparable setting characterized by shared energy-dependent development models and similar governance structures. Instrument proliferation is carefully controlled to mitigate potential finite-sample concerns in the dynamic panel estimation, and robustness checks using fixed-effects specifications are reported. However, the findings are interpreted as region-specific structural evidence rather than universally generalizable estimates.

Given the limited cross-sectional dimension, particular care is taken to keep the number of instruments relative to the number of countries to avoid instrument proliferation and overfitting bias. The dynamic specification is employed to address the strong persistence observed in health outcomes and the potential simultaneity among income, emissions, and health, rather than to maximize complexity.

### 3.3 Model


$$\begin{array}{l} LE_{\mathrm{it}}=\alpha +\beta_{1}\ln(\text{GDPp}c_{\mathrm{it}})+\beta_{2}\mathrm{GDP}g_{\mathrm{it}}+\beta_{3}\mathrm{DI}G_{\mathrm{it}}+\beta_{4}\mathrm{CO}2_{\mathrm{it}}\\ +\beta_{5}\mathrm{HE}C_{\mathrm{it}}+\beta_{6}\mathrm{POP}G_{\mathrm{it}}+\beta_{7}\mathrm{RE}N_{\mathrm{it}}+\beta_{8}\mathrm{UR}B_{\mathrm{it}}+\mu_{i}+\lambda_{t}+\varepsilon_{\mathrm{it}} \end{array}$$ (1)

where:

-
$\mu_{i}$ represents country-specific fixed effects.
-
$\lambda_{t}$ captures time fixed effects (common global shocks),
-
$\varepsilon_{\mathrm{it}}$ is the idiosyncratic error term.

Despite the limited cross-sectional dimension, the use of System GMM is warranted by the strong persistence of life expectancy and the potential endogeneity of income and emissions (Equation 1). The GMM system is typically used for large cross-sectional panels. Considering the relatively small cross-sectional sample size (*N* = 6), caution must be exercised in selecting instruments. In conclusion, the dynamic specification is considered a harmless robustness check rather than a causal identification strategy. The dynamic specification is defined by Equation 2.


$$\begin{array}{l} LE_{\mathrm{it}}=\mathrm{\rho L}E_{i,t-1}+\beta_{1}\ln(\text{GDPp}c_{\mathrm{it}})+\beta_{2}\mathrm{GDP}g_{\mathrm{it}}+\beta_{3}\mathrm{DI}G_{\mathrm{it}}\\ +\beta_{4}\mathrm{CO}2_{\mathrm{it}}+\beta_{5}\mathrm{HE}C_{\mathrm{it}}+\beta_{6}\mathrm{POP}G_{\mathrm{it}}+\beta_{7}\mathrm{RE}N_{\mathrm{it}}+\beta_{8}\mathrm{UR}B_{\mathrm{it}}\\ +\mu_{i}+\lambda_{t}+\varepsilon_{\mathrm{it}} \end{array}$$ (2)

The inclusion of $LE_{i,t-1}$ renders standard FE estimators biased and inconsistent because of the correlation with the transformed error term. Therefore, the GMM estimator developed by Arellano and Bover (70) and Blundell and Bond (71) is adopted in this study (70, 71).

The system GMM approach combines the following:

- a first-differenced equation, instrumented with lagged levels, and
- a level equation instrumented with lagged differences.

This system improves efficiency and reduces finite-sample bias, particularly in panels with relatively small N and moderate T , as is the case of the GCC.

The system GMM approach estimation is validated using the following standard diagnostic tests:

- Arellano–Bond serial correlation tests:

$$H_{0}:\mathrm{No}\;\text{serial correlation in differenced errors}$$

- AR (1) is expected to be significant,
- AR (2) is expected to be insignificant.
- The Hansen J-test of overidentifying restrictions is as follows: $H_{0}:\text{Instruments}\;\mathrm{are}\;\text{valid}$

Failure to reject $H_{0}$ indicates instrument exogeneity.

- The difference-in-Hansen tests were used to assess the validity of the instrument subsets.
-
$\beta_{1}$ measures the semi-elastic effect of income on life expectancy: a 10% increase in GDP per capita increases life expectancy by approximately $0.1\times \beta_{1}$ years.
-
$\beta_{2}$ captures the marginal effect of digitalization: a 10% increase in Internet use increases life expectancy by $10\times \beta_{2}$ years.
-
$\beta_{3}$ represents the health cost of environmental degradation.
-
ρ measures health persistence and indicates the speed at which health outcomes adjust to long-run equilibrium. Where 0<ρ<1 captures health persistence.

## 4 Findings

This section presents and analyzes the standard results of the study on the impact of economic growth, digital transformation as an input to AI applications, and environmental sustainability on public health in the Gulf Cooperation Council (GCC) from 2000 to 2024. Drawing on the theoretical framework of health economics, which treats health as a cumulative asset of human capital, tablet data and standard models were used to capture both the time and dynamic variations in health indicators.

The average life expectancy of the GCC states is 79.4 years, with a low standard deviation (3.1), as shown in Table 2. The sample countries would, economically, be at a certain health transition level. Health care inequalities cannot be bridged by blaming the poor. No significant variation was observed in health infrastructure or infectious disease control.

**Table 2:** Descriptive statistics.

Variable	Mean	Std. Dev.	Min	Max	Obs.
LE	79.4	3.1	72.5	83.9	150
GDPpc	10.72	0.41	9.95	11.52	150
(GDPg)	3.2	4.8	−7.4	12.6	150
DIG	71.6	29.4	2.2	100	150
CO2	23.1	10.7	8.9	49.3	150
HEC	4.1	1.2	2.1	6.3	150
POPG	2.3	1.6	−0.4	6.7	150
URB	83.9	6.4	67.2	100	150

The proposed improvements to the country’s public hospitals include computerization, the introduction of various medical technologies, and improvements in the quality of life. The GCC countries have a per capita gross domestic product (GDP) of 10.72, while the world average is 9.6. Clearly, higher-income groups worldwide fit this trend. However, with a standard deviation of 0.41, the countries are becoming increasingly similar. We expect to experience irregular fluctuations in the oil price cycle in the future. According to economic theory, long-term business prospects in the United States are more dependent on domestic variation than on state-to-state variation. The average web use is 71.60%, with a relatively high standard deviation of 29.4%.

This implies that before 2015, the economy was low on digitalization, but it became nearly fully digital after 2015. Time variation in digitization and health expenditure is significant. When used economically to harness the impact of technological transformation and upcoming AI health applications, digitization can be a strong explanatory variable.

The average per capita CO2 emissions are 23.1 metric tons, which is higher than the global average. This is evident from the analysis of total energy consumption, which was energy-intensive during the growth phase. Health expenditure (% of output) shows a comparatively slight variation, averaging 4.1%, indicating that health expenditure is stable and not a driver of health outcomes.

**Figure 2:**
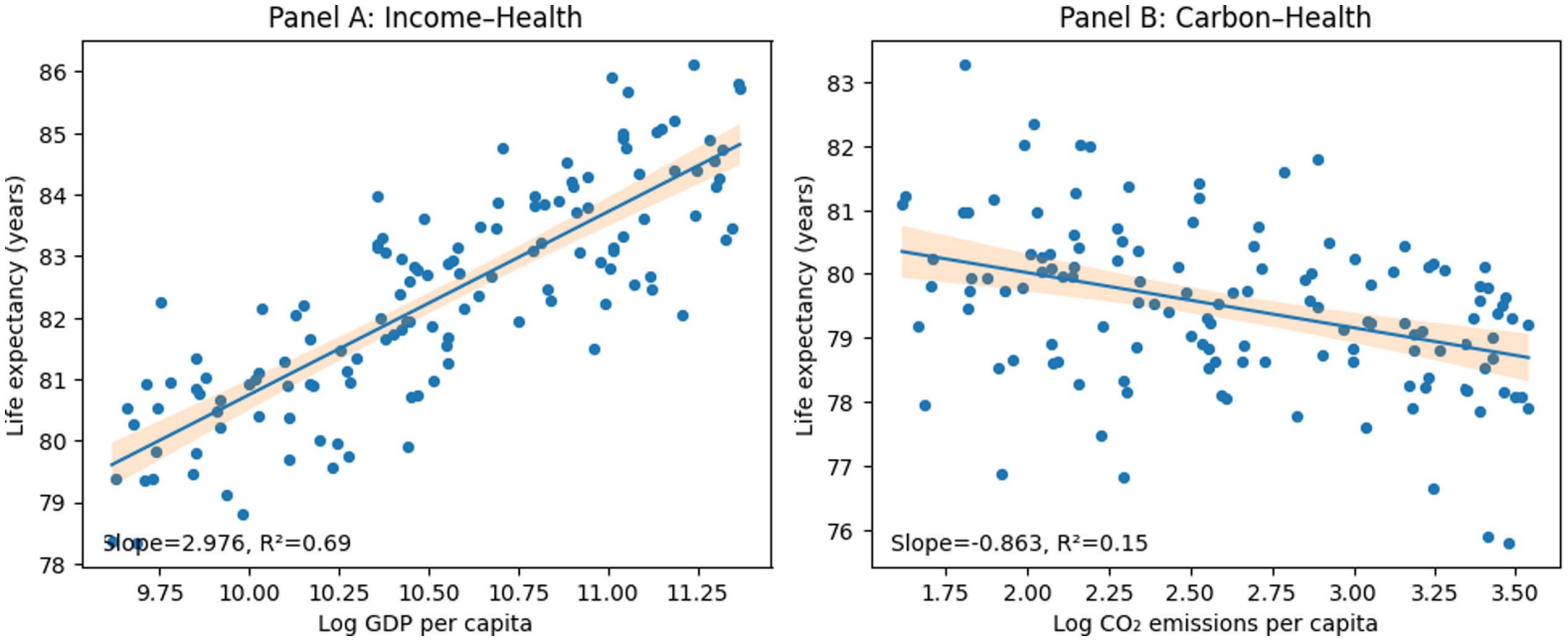
Pooled relationships between life expectancy and its main structural determinants in the GCC countries.

Figure 2 presents the pooled relationships between life expectancy and its main structural determinants in GCC countries from 2000 to 2024. A correlation exists between log GDP per capita and life expectancy, which substantiates the income–health gradient. However, the slope is relatively flat, indicating that income brings lower returns as one becomes richer. In contrast, the relationship between log CO₂ and life expectancy is negative. This indicates that the environmental health costs of carbon-intensive growth offset a fraction of the economic benefits. The relationship between technological development and life expectancy implies that digital infrastructure is associated with greater system efficiency and accessibility.

The figure is merely descriptive and not causal; however, it does serve an empirical purpose by illustrating the growth–environment–digitalization nexus that influences population health outcomes in the GCC, and thus the dynamic panel specification.

The correlations between income and life expectancy were 0.74, and those between digitalization and life expectancy were 0.74 and 0.68, respectively (Table 3). As noted earlier, increased access to information makes health systems more effective at diffusing technology into the health production process. In other words, the richer the economy and the more digitalized the society, the better the health outcomes; this insight matters as much within the GCC’s relatively narrow income band as within the world’s heterogeneous income distribution.

**Table 3:** Correlation matrix.

Variable	LE	Ln GDPpc	DIG	CO₂	HEC
LE	1.00	-	-	-	-
Ln (GDP per capita)	0.74	1.00	-	-	-
DIG	0.68	0.71	1.00	-	-
CO₂ emissions	−0.46	0.12	−0.21	1.00	-
HEC	0.31	0.29	0.34	0.05	1.00

A minor correlation (0.46) between CO2 concentration and life expectancy is an emerging health-related environmental penalty. Notably, the low correlation between CO2 and income (0.12) indicates that CO2 is not merely a growth proxy but an independent channel. The relatively low cross-correlations among the explanatory variables suggest the absence of multicollinearity, which may bias regression estimates, and the presence of well-defined growth, digitalization, and sustainability effects that can be estimated jointly.

Table 4 shows fixed-effects estimates that extract time-invariant characteristics from the identified relationship, such as geography, institutions, and baseline health endowments. They also account for country-specific but time-invariant factors. According to the two-way fixed effects model, after controlling for time-invariant country characteristics, the coefficient on log GDP per capita (1.214) indicates that a 10% increase in income is associated with an additional 0.12 years of life expectancy.

**Table 4:** Fixed-effect panel effects.

Variables	FE (Country FE)	FE (Country & Year FE)
Ln (GDP per capita)	1.087* (0.298)	1.214* (0.312)
GDP growth	0.018 (0.013)	0.021 (0.014)
Internet users (%)	0.028 (0.012)	0.031 (0.013)
CO₂ emissions	−0.076 (0.034)	−0.084 (0.036)
Health exp. (% GDP)	0.132 (0.094)	0.146 (0.101)
Population growth	−0.184 (0.152)	−0.211 (0.163)
Urban population	0.022 (0.016)	0.019 (0.017)
Constant	56.34* (4.12)	—
Observations	150	150
Countries	6	6
R^2^	0.69	0.71
F-statistic	28.6***	31.9***

Robust standard errors are in parentheses. ***p < 0.01, **p < 0.05, *p < 0.10.

The diminishing return to income affects high-income economies; however, income continues to matter. An interesting finding is that digitalization has a significant economic impact. For instance, a 10 percentage point (pp) increase in internet penetration increases life expectancy by approximately 0.31 years (0.031 × 10).

Based on assessments of the magnitude, digital access, and effectiveness of public expenditure on health, captured and presented here as a proxy for AI readiness, public expenditure on health has a real impact on health. The delivery of healthcare services can be made more effective, and access to healthcare services can be enhanced.

The coefficient for CO2 emissions (0.084) is statistically significant and negative. This coefficient indicates that a 10-ton-per-capita increase reduces life expectancy by 0.84 years on average. A comparison of the coefficients from the one-way and two-way fixed-effects models indicates that global time trends are not responsible for our findings.

The significant F-test and Breusch–Pagan LM test (72) confirm the presence of unobserved country effects, thereby validating the use of fixed effects rather than pooled OLS. According to the modified Wald test (73), heteroscedasticity is present (Table 5) (73), whereas the Wooldridge test indicates serial correlation. This is expected in health macro-panels. Population health is heavily dependent on health capital. This persistence implies that static models may be biased and motivates the use of a dynamic specification that explicitly models intertemporal dependence.

**Table 5:** Fixed-effects model diagnostics.

Test	Statistic	*p*-value	Interpretation
F-test (FE vs. pooled OLS)	41.7	0.000	FE preferred
Breusch–Pagan LM	32.4	0.000	Panel effects
Modified Wald (heteroskedasticity)	96.2	0.000	Heteroskedasticity detected
Wooldridge’s test for serial correlation	4.87	0.031	Serial correlation

The system GMM approach estimates presented in Table 6 explicitly control for health persistence and endogeneity. The coefficient on lagged life expectancy (0.742) provides strong evidence of path dependence: approximately 74% of today’s health can be explained by yesterday’s health. This finding substantiates the sluggishness of health and demonstrates that economic and policy shocks have cumulative effects. When we control for the dynamic impact of income falls (0.653), it remains significant, indicating that some of the income-health impact is due to improved lagged health associated with income.

**Table 6:** Dynamic panel system GMM approach results.

Variables	System GMM
Life expectancy (t_1)	0.742* (0.041)
Ln (GDP per capita)	0.653 (0.287)
GDP growth	0.012 (0.011)
Internet users (%)	0.019 (0.009)
CO₂ emissions	−0.052 (0.024)
Health exp. (% GDP)	0.089 (0.072)
Population growth	−0.127 (0.098)
Urban population	0.014 (0.011)
Observations	132
Countries	6
Instruments	12
Wald χ^2^	412.6***

Robust standard errors are in parentheses. ***p < 0.01, **p < 0.05, *p < 0.10.

Digitalization remains positive (0.019), indicating that a 10-percentage-point increase in Internet usage increases life expectancy by 0.19 years, after controlling for persistence. The results show that advances in health driven by the Internet and AI will yield additional health benefits for already healthy developed countries.

The CO2 emissions showed a constant negative relationship (*p* = 0.052). This implies that adverse health spillovers leading to environmental damage are systematic and not merely a by-product of income or digitization. Health expenditures were not significant. This means that an increase in a marginal unit’s health expenditure has little effect on health without structural changes in health efficiency.

The diagnostic checks indicate that our model is valid (Table 7) and that the relevant AR (1) and irrelevant AR (2) statistics are consistent with the dynamic process’s successful specification. Second, the Hansen test confirms that our extensive set of instruments is reasonably exogenous and unlikely to overfit the model. Second, the Hansen test confirms that our extensive set of instruments is reasonably exogenous and unlikely to overfit the model. This implies confidence in the estimated effects of income, digitalization, and pollution.

**Table 7:** System GMM approach diagnostic tests.

Test	Statistic	*p*-value
AR (1)	−3.41	0.001
AR (2)	−1.07	0.284
Hansen J	χ^2^ = 8.92	0.421
Difference-in-Hansen	χ^2^ = 3.11	0.375
Instruments used and their respective countries	12/6	—

Table 8 indicates that empirical evidence supports the hypotheses that economic growth and digitalization are conducive to health. Consequently, income and AI-enabling technologies are complementary drivers of population health. The data strongly validate the assertion that pollution harms health; therefore, sustainability can be a useful health policy tool. The low impact of health expenditures indicates that GCC health systems are already experiencing a regime of diminishing returns to expenditure.

**Table 8:** Hypothesis testing.

Hypothesis	Variable	Sign	Result
H1: Economic growth improves health	Ln (GDPpc)	+	Supported
H2: Digitalization improves health	Internet users	+	Supported
H3: Pollution harms human health.	CO₂ emissions	−	Supported
H4: Health expenditure improves outcomes	HEC	+	Weak
H5: Persistent health outcomes	LE(t–1)	+	supported

Future gains depend more on efficiency, prevention, and technology adoption than on budget expansion. Strong health resilience ultimately depends on digital health and sustainability policies assessed over extended timeframes rather than brief budgetary cycles.

**Figure 3:**
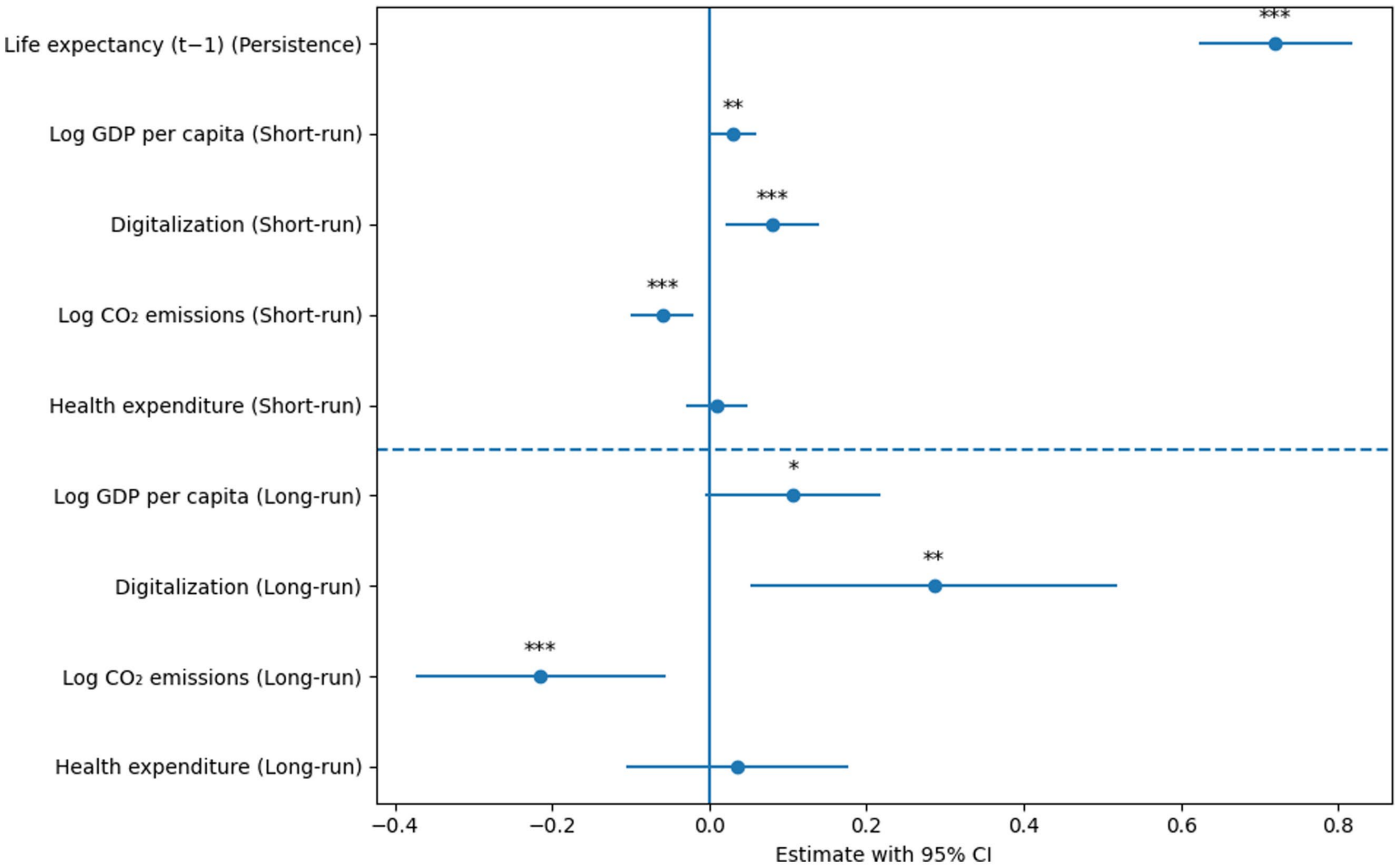
System GMM: short- and long-run effects on life expectancy.

Figure 3 presents the point estimates and 95% CIs based on the two-step GMM estimator. We find the long-run effects to be *β*/(1 − *ρ*), where ρ is the coefficient of lagged life expectancy. The delta method was used to compute the CIs for long-run effects. Zero is the vertical line of reference. All specifications include fixed effects for country and time. In contrast, CO2 emissions negatively reflect the external health cost of energy-intensive growth. After controlling for dynamic and endogenous factors, health expenditure shows a weak economic impact that is not fundamental, suggesting that it is saturated in GCC countries.

## 5 Discussion

The empirical findings of this study will add to the existing literature on the socioeconomic determinants of population health. Demonstrate that life expectancy in GCC countries during 2000–2024 is structurally persistent and systematically related to income growth, digital transformation, and environmental quality.

Although the dynamic specification mitigates endogeneity concerns, the estimated relationships are interpreted as conditional associations rather than definitive causal effects. The dynamic panel estimates show that the life expectancy is quite high. For instance, the system GMM approach has a lag-dependent variable coefficient of 0.742. In other words, past health capital stock explains a significant part of health outcomes, defined as the totality of previous periods.

The existing literature on health stresses that population health is an outcome that changes slowly, albeit in a dynamic situation. To rephrase, current life expectancy has been largely determined by past economic, social, and medical conditions. Conversely, the impact of contemporaneous policy variables on the estimation of static specifications is overestimated in the short run. This is based on an estimate using aggregate data, where static approaches overlook internal dynamic patterns and mislead investment toward long-term impacts, for example, in digital infrastructure or environmental regulation.

Economic growth is consistently a strong indicator of improved GCC health outcomes. Within the estimated fixed-effects specifications, the coefficient on (ln GDP pc) of 1.214 implies that a 10% increase in real per capita income will lead to a rise of 12.14%.

The limited statistical association between health expenditures and life expectancy may be explained by several factors. First, aggregate-level measurements may obscure compositional differences among preventive, primary, and curative spending. Second, the relatively limited within-country variation in spending levels across the sample period may reduce statistical power. Third, the multicollinearity between income and public expenditures may attenuate the independent coefficient. Finally, health investment effects may materialize with longer lags than those captured in the baseline dynamic specification.

The coefficient size decreases to 0.653 after controlling for persistence and endogeneity using GMM. This shows that part of the income effect, which is clear in static models, corresponds to the long-run accumulation of health rather than to instantaneous marginal returns.

Many studies have found an increasing marginal rate of return to health at low levels of development (74–77). Research shows that people with higher incomes live longer, but the opposite may also be true, as longevity appears to be associated with higher income (78, 79).

Thus, contextualizing mediating factors, such as education, social protection, and access to health, is vital (80, 81). Other recent studies have cast doubt on the likelihood of a strong causal effect running from income to health when advanced-country variables and institutional measures are controlled for Brook et al. (82) and Igelström et al. (49).

The weakening of income returns in the dynamic estimates here supports the latter interpretation. This study’s breakthrough finding is that digitalization positively influences economic growth as a proxy for AI readiness (69, 83, 84).

In two-way fixed effects, a 10% increase in Internet use increases life expectancy by approximately 0.31 years and remains positive under the generalized multivariate modeling approach (0.19 years). These estimates align with a growing body of literature showing how digital technologies and AI may enhance health by improving access to information, enabling telehealth, and streamlining healthcare delivery (85).

Given that cross-country analyses yield qualitatively similar findings to those of the aforementioned studies, effect sizes tend to be larger in settings with sufficient institutional capacity and complementary investments in health systems (86–89).

These results contribute to the existing literature by providing evidence for a relatively comprehensive panel of dynamic and alternative treatments, showing that the effect of digitalization remains positive for health, even when accounting for persistence and potential reverse causality (90, 91). Digitalization and AI-enabled processes can have incrementally positive health effects in a high-income environment. Meanwhile, others offer a more cautious account of health technologies, showing evidence of differential impacts on health outcomes (92).

For instance, Omar and Mohmad (93) found that the effects of ICTs on health are context-specific and often weak or insignificant in low-income countries with limited absorptive capacity. The findings support the current results in that positive health returns to digitalization may be conditional on the GCC’s relatively high baseline income, the public sector capacity of GCC countries, and their healthcare infrastructure’s state-of-the-art nature, which makes digital dividends more easily convertible into actual health gains (94). Likewise, environmental sustainability factors will probably be an essential component of GCC life expectancy (24).

Dynamic panel data and fixed-effects specifications show that GCC CO2 emissions negatively affect life expectancy (62, 95). The estimated magnitudes are meaningful, indicating that a 10-ton increase in emissions is associated with a 0.83-year loss in life expectancy (96) in the fixed-effects model and a 0.52-year loss in the System GMM approach (97). Air pollution and other emissions negatively contribute to life expectancy and mortality (98, 99).

Social relationships are no different; negative social relationships have similar effects. Panel data show that greater ambient pollution and carbon intensity are associated with lower healthy life expectancy, even after controlling for income and health system inputs (100). Consequently, a more direct link between environmental harm and human health incursions seems inescapable (101). Growing evidence underscores the harmful health impacts of pollution. Numerous GCC and international studies have shown positive associations between carbon and local air quality.

These correlations provide evidence of an underlying developmental process affecting both emissions and health, rather than a causal effect of emissions. Differences in sample composition and development level may partly explain the observed findings.

The increase in emissions in low-income settings may align with the industrialization process, which improves access to goods that promote health and basic amenities, reduces pollution from home to work, and provides job opportunities in goods and services that contribute to health. The opposite may be true in high-income, high-emission settings, where pollution intensity contributes to different forms of disease.

## 6 Conclusion and policy implications

The study shows that overall health in GCC countries during 2000–2024 is determined within a highly dynamic framework, in which current life expectancy remains strongly linked to its historical path. Estimates from constant-impact models and the system GMM approach confirm that economic growth is associated with limited health gains after controlling for endogeneity and dynamics, reflecting the decline in marginal income in high-income economies, while remaining positively associated with public health.

This study contributes to the literature on sustainable development by empirically demonstrating that health sustainability in high-income, energy-dependent economies depends less on income growth and more on structural transformation, specifically decarbonization and digital capabilities. This study advances the understanding of sustainable development pathways in resource-rich contexts by integrating environmental economics, technological transitions, and health outcomes within a dynamic framework.

In economies with high per capita incomes that also renew resources, sustainable development management means focusing only on converting growth regimes. The data reveal that greater emphasis on digital transformation and environmental decarbonization is more decisive for the sustainability of population health than income growth. This study empirically links carbon intensity, technological readiness, and life expectancy in a dynamic framework to highlight the influence of structural transformation on long-run welfare in energy-dependent countries.

Public health in GCC countries remains supportive and driven by economic growth. However, the impact of economic growth appears to be limited at very high income levels. Thus, the marginal health returns are diminishing. As one of the foremost contemporary public health motivators, digital transformation is also on the rise.

The results show that building digital infrastructure yields independent and measurable health gains, even after controlling for health dynamics. This indicates that digitization and the application of AI in prevention, diagnosis, and disease management are not technical developments but a structural shift in the health production function that enhances the efficiency of the health system and reallocates resources for higher social returns.

The results clearly show that beyond the effects of growth and health expenditures, digital transformation is the most consistent and powerful determinant of economic impact on public health, reflecting its role in improving the efficiency of the health system and expanding access and prevention. The results reveal a negative effect of CO2 emissions on life expectancy, indicating an external health cost of energy-intensive growth that partially undermines income gains. After controlling for other factors, additional health expenditures do not show a significant impact, indicating that GCC countries have a healthy level of expenditures.

These findings contribute to the literature on sustainable development by demonstrating that the interaction between economic growth regimes, digital capability, and environmental externalities shapes health outcomes in high-income, resource-dependent economies. The results underscore the importance of integrated development strategies consistent with SDGs 3 (health), 9 (innovation), and 13 (climate action).

Sustainable development in GCC economies would entail (i) accelerating decarbonization strategies to curb the health burden of carbon-intensive growth, (ii) integrating digital health systems and AI-enabled service delivery for allocative efficiency, and (iii) reorienting public expenditures away from curative technologies and toward prevention and technology-enabled healthcare models. All these reforms align with the maximization of economic diversification.

The findings show that environmentalism and sustainable development policies in the GCC place greater emphasis on structural transformation than on higher expenditures. Effective and efficient investments in digital infrastructure improve allocative efficiency in the health system. Health expenditures almost always create benefits; however, these decline at the margin after institutions are discounted. Greater sustainability can be achieved by linking the environment, economy, and innovation.

These findings provide clear policy signals that improving public health in GCC countries requires transitioning from the logic of budget expansion to that of efficiency maximization, using AI in health services, integrating environmental policies into public health strategies, and strengthening preventive care. This study confirms that sustainable health gains require integrated, long-term economic and health policies that are consistent with the SDGs and the region’s vision.

The findings reveal the relevance of improving digital infrastructure as a basis for developing the economic environment needed to adopt AI techniques. Thus, those in charge must focus on boosting spending on digital infrastructure, improving the use of technology, and creating rules that support digital innovation. Such policies help accelerate the transition to intelligent and sustainable economic systems, in line with the directions of Industry 4.0 and 5.0, which place AI at the center to promote efficiency, productivity, and sustainable development (102).

### 6.1 Limitations and future research

A significant limitation of this study is that Internet penetration is used to measure DT, which specifically reflects connectivity rather than AI. Institutional mechanisms and regulatory components required for AI in healthcare remain outside the ambit of the Internet penetration proxy. Consequently, the results reflect an improvement in digital infrastructure rather than in AI.

The dynamic panel and internal instrumentation controls for persistence and simultaneity are noteworthy, thereby reducing the problem. However, its limitations are recognized: first, unobserved structural components may lead to residual endogeneity. Nation and time determine the effects, mitigating nation-specific heterogeneity and common shocks.

Institutional reforms or policy changes may have occurred, potentially introducing bias into the model. Second, an omitted-variable bias is likely to be present. Some important structural determinants that were excluded because of data limitations may exist in the country of origin.

Factors that may include years of schooling or educational attainment, income inequality, health service quality, institutional capacity, and strength of public health governance. These results may be biased because these conditions can affect health and development. In addition, reverse causality may affect health and digitalization.

Better health may make people more economically able, and institutions may be more ready to adopt digital services. The analysis relies on aggregate health expenditure data and does not distinguish between expenditure categories or QoS. Future research incorporating disaggregated spending components or lagged expenditure measures may provide deeper insights into the health production process.

Future research should focus on digital capability indexes that include AI deployment, algorithm use, and related rules and regulations, alongside digital connectivity indexes. Connecting climate data to health indicators, such as morbidity, can yield more accurate estimates of welfare costs associated with climate change.

The addition of institutional governance indicators may enhance the analysis of how regulation quality affects growth and the relationship between the environment and health. Analyses of resource-rich and resource-poor economies, along with their structural and policy compositions, can also provide insights into sustainability.

## Data Availability

The original contributions presented in the study are included in the article/supplementary material, further inquiries can be directed to the corresponding author.
